# Distribution of Mechanical Properties of Steel Along the Curvature of Corrugated Web SIN Girders

**DOI:** 10.3390/ma19040791

**Published:** 2026-02-18

**Authors:** Witold Basiński, Grzegorz Gremza

**Affiliations:** Faculty of Civil Engineering, The Silesian University of Technology, Akademicka 5, 44-100 Gliwice, Poland; grzegorz.gremza@polsl.pl

**Keywords:** corrugated web, coefficients of variations, Gaussian distribution, strength, yield strength (point), permanent strain, residual stress, hardness

## Abstract

**Highlights:**

**What are the main findings?**

- The study presents the results of statistical investigations into random strength parameters of steel used in manufacturing of corrugated webs for SIN plate girders, depending on the place of the specimen cut-out, that is, from the flat section or ridge of the wave. Metallographic and Vickers hardness tests were performed. Differentiation of mechanical properties was confirmed. Values of deformations and residual stresses were determined.

**What are the implications of the main findings?**

- The performed tests demonstrated close relationships between the effects of web metal and strength parameters along the web curvature on the beginning of the failure phase of the corrugated web while buckling was initiated. Such conclusions are made which can be useful for design purposes.
- This provides insights for future research on sinusoidal web stability.

**Abstract:**

This paper presents results from statistical tests on random parameters of strength properties of steel used to manufacture corrugated webs for SIN plate girders, depending on the place of specimen cut-out, that is, from the flat section or ridge of the wave. The tests were performed on specimens collected from 12 randomly selected corrugated sheets with thicknesses of 2, 2.5 and 3 mm, provided by the manufacturer of SIN beams. The analysis was used to select variation coefficients of yield strength *V*_Re_ = *D*(*R_e_*)/*E*(*R_e_*) and partial coefficients of yield strength *γ_m_* for steel in flat and arched parts of the web. Metallographic and Vickers hardness tests were performed. Values of deformations and residual stresses were determined. The close correlation between the influence of the web plate shape and the strength parameters along the web curvature was demonstrated. Analysis of the initiation points of stability loss (IPLS points) revealed that the initiation of stability loss occurs in the area of the flat web sections. In addition to the influence of geometry, the influence of the change in yield strength, as identified in this paper, can be observed. Consideration of random features of yield strength and web thickness can lead to modifications in designing and calculating structures made of SIN girders.

## 1. Introduction

**Figure 1:**
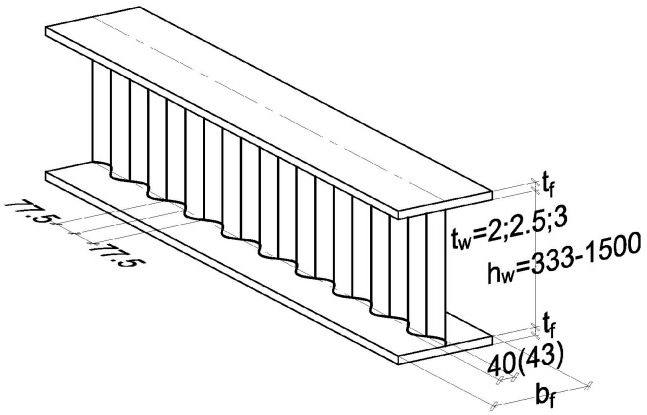
SIN–type corrugated web girder.

After corrugated sheet was patented by H. Palmer in 1829 as a formed sheet of thin steel, technology for using it as a structural element was developed. In the 1930s, it was found that corrugations of sheets could be used as web stiffeners to increase the web stiffness and critical shear stresses. This began the process of using corrugated sheet metal for webs in plate girders. In currently produced girders with SIN corrugated web [1], a wave-formed sheet with a span of 2×77.5 = 155 mm and three standard thickness values, *t_w_* = 2.0, 2.5, and 3.0 mm (Figure 1), are used for webs. The height of available girders ranges from 333 to 1500 mm at a maximum length of pre-assembled units up to 16 m. According to [1], the overall height of the wave is *h_s_* = 40 mm at thickness *t* = 2 mm and *t* = 2.5 mm, and *h_s_* = 43 mm at thickness *t* = 3 mm. A wave consists of three segments, a circular segment in the approximation on the ridge and two straight segments corresponding to the sinusoidal shape at an amplitude *f* = 20 mm (for *t* = 2 mm and *t* = 2.5 mm) or *f* = 21.5 mm (for *t* = 3 mm).

**Figure 2:**
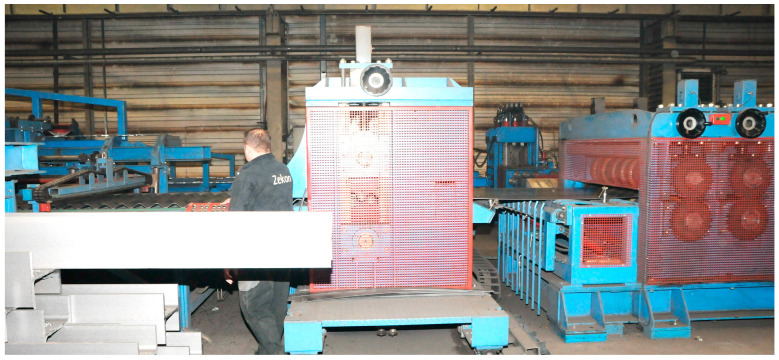
The process of bending sheet metal to a sinusoidal shape (in the middle, the rolling mill; on the right, the straightening machine; and on the left, the corrugated strip).

The production process of corrugated webs affects strength parameters of steel, and also resistance of the whole girder. The overall production process of the corrugated web from steel begins at steel works and includes the following stages:

(1) Slab casting with a thickness of 220 mm in the steel plant;
(2) Rough rolling of the slab;
(3) Hot rolling of sheet until required thickness: 2; 2.5; 3 mm;
(4) Cooling down and winding flat plate onto a coil with an internal diameter of 740 ÷ 760 mm;
(5) Rolling out;
(6) Cold straightening;
(7) Longitudinal cutting;
(8) Cold forming of corrugated web at a specially prepared eye rolling mill (Figure 2).

In the last stage, flanges from universal plate are welded into the web to form a girder with sinusoidal corrugated web (Figure 1).

**Figure 3:**
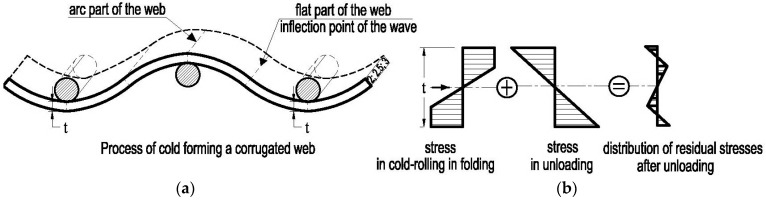
Stresses arising along the wave in the process of forming a corrugated web: (**a**) arc and flat parts of the web created during cold–forming process, (**b**) residual stress formation.

Cold forming provides the final shape of the web with an arched segment on the wave ridge and the flat part at the wave inflection (Figure 3a). In addition to structural changes observed during cold formation, strength parameters of steel obtained during tensile test also had an impact on the system of residual stresses created in the corrugated web due to cold formation along the wave (Figure 3b). Hence, it is reasonable to determine the impact of the production process of corrugated web on strength parameters of steel and differences in mechanical parameters along the web curvature on the flat and arched parts of the wave.

**Figure 4:**
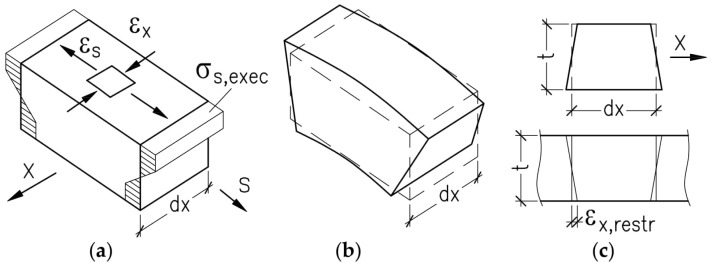
The formation of restrained deformations: (**a**) stress during formation along the wave in the s direction, (**b**) free deformation, and (**c**) deformation restrained in the x direction.

Apart from residual stresses in the direction of bending, the stress state is also formed in the direction of the generating line (the direction s)—Figure 4. This state must be caused by constraining side extension of material at its elongation or shortening (Figure 4c). Assuming that rotation of the cross-section directly on the surface x is impossible (the flat state of deformation formed during the formation), the restrained value of permanent deformations at the web surface area can reach approximately the value *ε_x,restr_ = υ·ε_Res,s_*, where *υ* is Poisson’s ratio.

Complex processes occurring inside the material and significant deformations that develop throughout the production process hinder the definite assessment of free-stress state based on analytical or numerical calculations, particularly in the direction perpendicular to the direction of rolling (direction x in Figure 4). However, it is possible to make a joint assessment of impacts of free stresses and material hardening at bending on the average strength of material in the finished web, which is used in calculations. This assessment was made by analysing results from simple tensile testing described in this paper.

The guaranteed yield strength of delivered hot-rolled flat sheet from steel grade S235, known as the specified minimum, is *R_emin_* = 235 MPa [2]. The value R_emin_ is determined from tests on steel specimens prior to corrugation after stage 4, and steel with yield strength below 235 MPa is rejected on the basis of these tests. Extensive statistical studies on strength of metallurgic products made of structural steel were performed by Sowa, Murzewski, and Mendera [3,4,5]. Similar statistical studies were made in Czechia [6,7,8] and in Germany [9]. The mechanical properties of 0.5–2 mm thin sheets were verified by Gwóźdź, Woźniczka in 2005–2010 [10]. They presented ranges of yield strength of sheet steel with thickness suitable for producing corrugated webs.

The distribution of the yield strength of steel was prepared and variation coefficients of yield strength *V_Re_* were determined. These were used to estimate partial coefficients of yield strength *γ_M_*. Based on the tests and according to [11], nowadays the manufacturer of girders provides the yield strength *R_e_* = 235 MPa for corrugated webs. The available design guidelines and standards [1,12,13,14] do not contain provisions to include the favourable effect of cold folding [15] on strength parameters along the web curvature. Considering residual stresses, only the effect of welding stresses on the resistance of girders with trapezoidal and flat webs was analysed [16,17,18]. Previous research conducted in the USA and Canada [19,20] focused on the application of different types of waves and trapezia on resistance of metal coats, but they only linked parameters of the modulus of Young and Kirchhoff, ignoring a change in strength parameters of steel. Recent works usually focus on effects related to shear resistance of corrugated webs [19,21,22,23,24,25]. The initiation of buckling of corrugated webs was observed to be concentrated in flat segments of the web wave [26,27]. It should be emphasized that the initiation of web buckling is significantly affected by random variability in material strength, and its change along the wave curvature.

Only the standard EN 1993-1-3 [28] provides the design-related increase in yield strength of the material related to the number of bending points of the sheet. In the case of corrugated webs verified in accordance with the standard EN 1993-1-5 [13], the changes in mechanical properties along the wave curvature and the effect on web buckling are not analysed.

Hence, the tests were conducted on the mechanical properties of steel in corrugated webs including their change along the wave curvature. The tests were conducted on 144 randomly selected specimens taken from twelve sheets of corrugated webs, with six specimens from each thickness: 2, 2.5 and 3 mm taken from flat and arched parts. Based on the performed tests, the variable yield strength *R_e_* and tensile strength *R_m_* were estimated, to which variation coefficients of yield strength *V_Re_* and strength *V_Rm_* were assigned with corresponding partial coefficients *γ_Me_* and *γ_Mm_.* In addition, the values of Young’s modulus *E* were determined. The analysis of strength tests and tests on material structure demonstrated the variability in the strength parameters of steel along the curvature of a web wave, which have an impact on points initiating buckling of the corrugated web.

## 2. Strength Tests on Corrugated Webs

**Figure 5:**
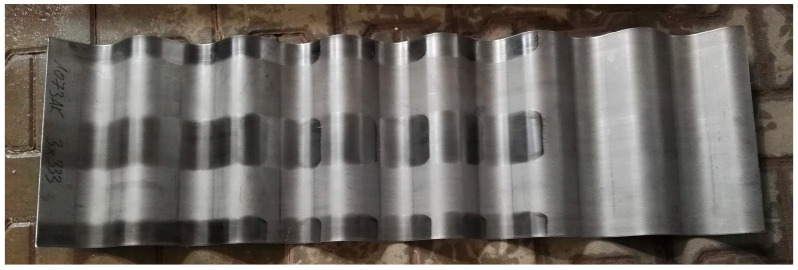
Sheets for corrugated webs.

Specimens for material tests were taken from 12 metal plates from a one-time shipment of the corrugated webs (Figure 5) which were not used by the SIN girder manufacturer in his final product. Flat iron sheets without mechanical treatment were not tested due to the lack of supply from the web manufacturer.

Dimensions of metal plates (Table 1) were 333 × 1085 mm, which means that their height corresponded to the available height of a web in the SIN girder, and simultaneously was a multiple of the wave length (7 × 155 mm).

**Figure 6:**
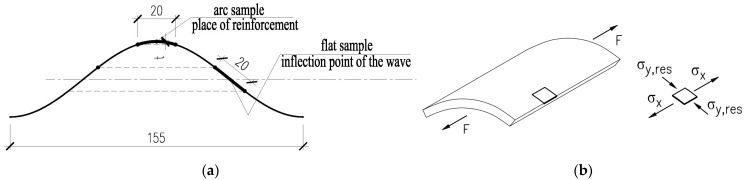
Wave shape: (**a**) the place where the samples were cut from the corrugated web along the fold; and (**b**) the direction of loading F and arrows indicating stress applied during test in direction x and residual stress in direction y.

For each obtainable thickness of the corrugated web, 2, 2.5, and 3 mm, twelve sheets were prepared, four for each thickness. Then, standard specimens were waterjet cut along the corrugation from the prepared sheets (Figure 6). Samples were taken from pieces of corrugated webs only from the basic corrugated sheet without the influence of welding.

As strength parameters of the web can change following the sinusoidal change in the web geometry, six specimens were taken from the flat and convex part of each sheet (Figure 6).

**Figure 7:**
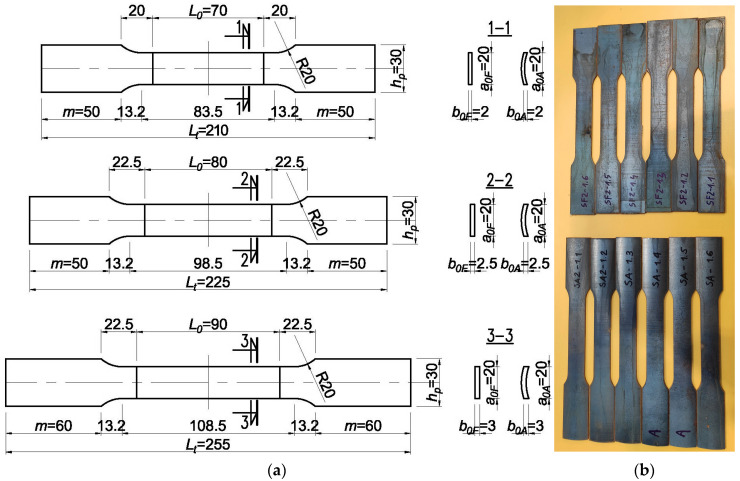
Flat and curved samples: (**a**) dimensions of samples from a web with thicknesses of 2, 2.5, and 3 mm; (**b**) flat and curved samples after cutting.

The distribution of mechanical properties of steel along the curvature of the corrugated web was tested on specimens cut out as specified in the standard [29]. Corrugated webs delivered for the tests were made from nominal steel S235JRG2. The specimens of “10-fold” base were cut out from corrugated sheets (Figure 7). Geometric dimensions of the specimens were verified using a calliper with a vernier scale of 0.1 mm. In total, 144 specimens were tested, including 24 specimens from each nominal thickness, 2 mm, 2.5 mm and 3 mm, cut out from the flat part, and 24 specimens from each nominal thickness, 2 mm, 2.5 mm and 3 mm, cut out from the arched part of the sheet. The test results were affected by both the direction of hot rolling and cold folding of the sheet in the process of producing corrugated web.

**Figure 8:**
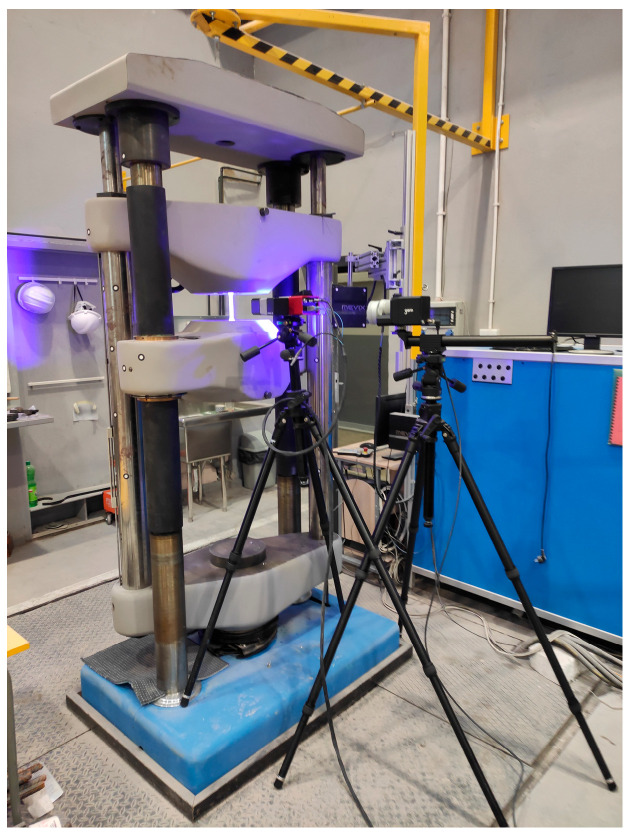
Test stand with a system of digital image correlation (DIC).

The distribution of the mechanical properties of steel along the curvature of the corrugated web was tested using the testing machine PUL 400 VEB Werkstoffprüfmaschinen Leipzig manufactured in the GDR, modernised by LaborTech (Czechia) and the Aramis system—digital image correlation (DIC) (Figure 8). The Aramis system was calibrated using the calibration panel with a length of 243.265 mm and deviation of 0.058 pixels (optimized 0.009 pixels).

The Aramis system was used to take measurements during the tests of the tensile strength P and elongation ΔL of the specimen datum L_0_ (Figure 8). The increment rate of tensile strength during the tests did not exceed 0.4 kN/s. Mean values of yield strength Re, tensile strength Rm, extensibility A_10_, and Young’s modulus, estimated to be within the range from 20 to 80% of the elastic domain, are presented in Table 2 and Table 3. To estimate strain values used in Young’s modulus assessment, virtual strain gauges in the GOM Correlate v.18 (GOM Software) program were defined.

**Figure 9:**
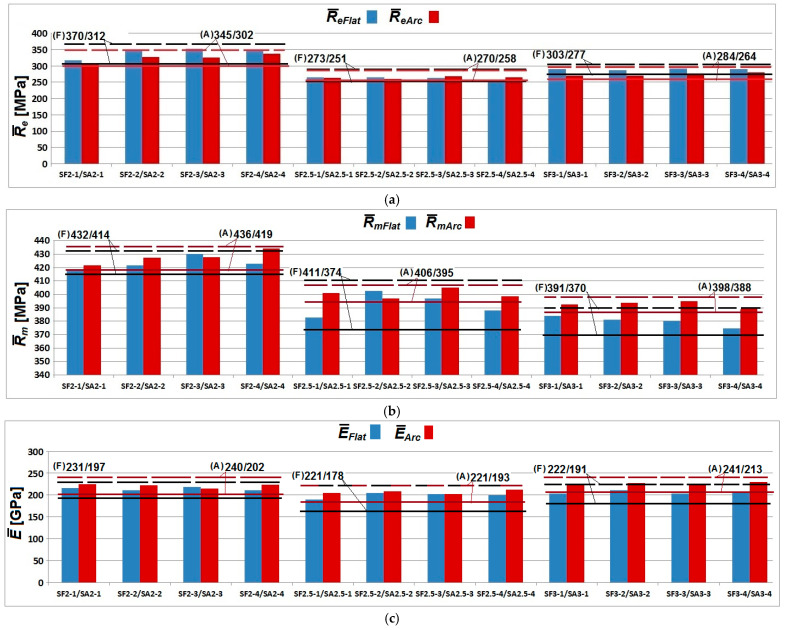

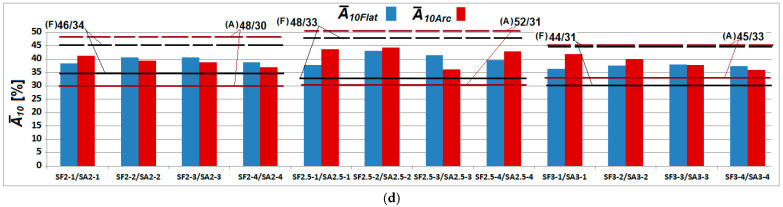
Summary plots showing distributions of *R_e_*, *R_m_*, *E*, and *A_10_* for flat vs. arched samples of three thicknesses 2 mm, 2.5 mm and 3 mm with confidence intervals: (**a**) yield strength *R_e_* ; (**b**) tensile strength *R_m_*; (**c**) Young modulus; and (**d**) elongation *A_10_*.

Summary data from Table 2 and Table 3 for *R_e_*, *R_m_*, *E*, and *A* are presented in Figure 9a–d. Confidence levels corresponding to the 5% safety quantile according to EN 1990 [30] are indicated.

**Figure 10:**
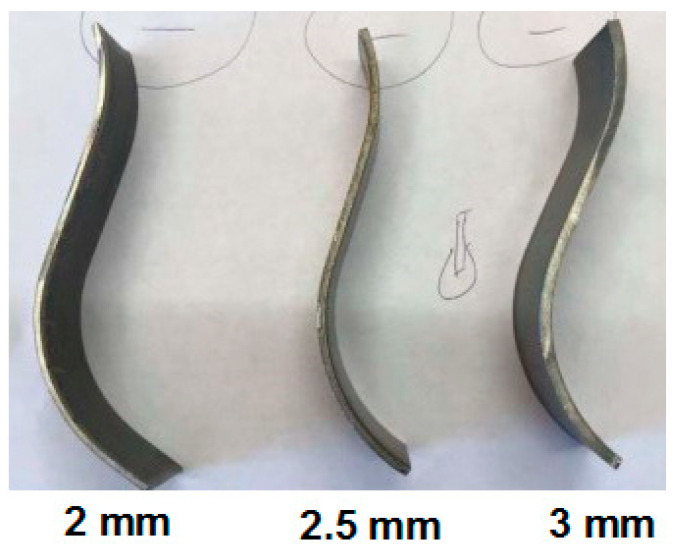
Side view of samples with different thicknesses (from left): 2, 2.5 and 3 mm cut from a corrugated web.

To determine the structure of steel from tested webs, microscopic metallographic examinations were conducted. The examinations were performed on polished sections taken from the flat part and the arched part of the web (Figure 10).

**Figure 11:**
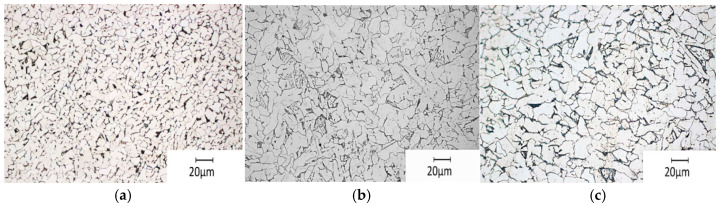

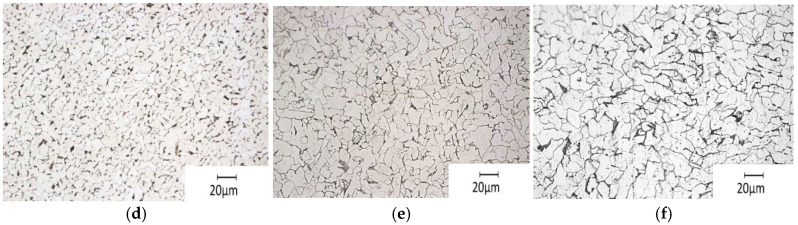
Microstructure of the tested Nital-etched samples, sampled from the 2, 2.5 and 3 mm webs: (**a**–**c**) arch area and (**d**–**f**) straight area, magnification 500×.

A Zeiss optical microscope, using the bright field observation technique, was employed in the tests. Polished sections were prepared by grinding with sandpaper and polishing with a diamond suspension. The microstructure of materials taken from the examined specimens was evaluated on the basis of Nital-etched polished surfaces. Examples of test results presented as metallographic photos for individual specimens are illustrated in Figure 11.

The performed tests demonstrated that elements of the corrugated web were made from steel with a ferrite-perlite structure (with a minor contribution from perlite). The perlite content was estimated as small. They did not show a clear difference in the material structure between the flat and arched areas. The arrangement of grains in polished surfaces of the specimens taken from the corrugated webs, which were used to examine the distribution of mechanical properties of steel along the curvature for each thickness, 2, 2.5, and 3 mm, was regular and had a heterogeneous material structure (Figure 11). The permanent deformation of a few percentage points was too small to cause noticeable changes in the material microstructure. Only a difference in grain size caused by the thickness of the web sheet was clear. Due to lower deformation values compared with typical cold–formed beam section corners, grains were not visibly elongated.

The known correlation between the grain size and the average tensile strength was confirmed. It was similar in the specimens with a thickness of 2.5 and 3 mm and with similar grain size, and considerably higher in the specimens with a thickness of 2 mm and finer grain.

**Figure 12:**
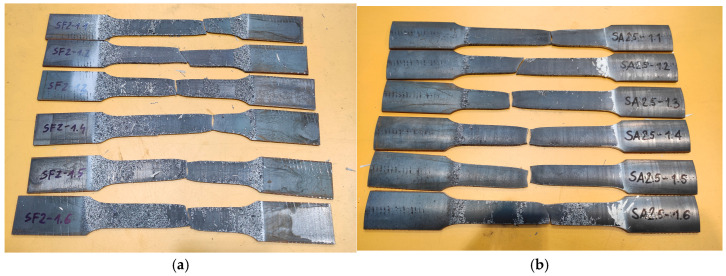
View of tested samples: (**a**) 2 mm flat sample and (**b**) 2.5 mm arch sample.

The tests were used to draw diagrams for the relationship σ–ε for all flat and arched specimens. Photos of tested specimens are shown in Figure 12.

**Figure 13:**
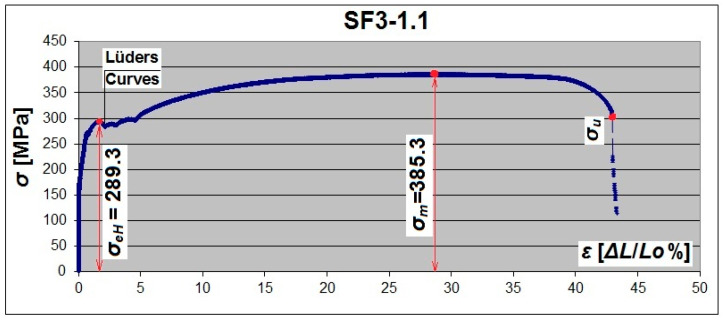
Tensile σ-ε diagram of a 3 mm thick flat steel sample with a corrugated web (based on an additional optical extensometer used with the testing machine).

**Figure 14:**
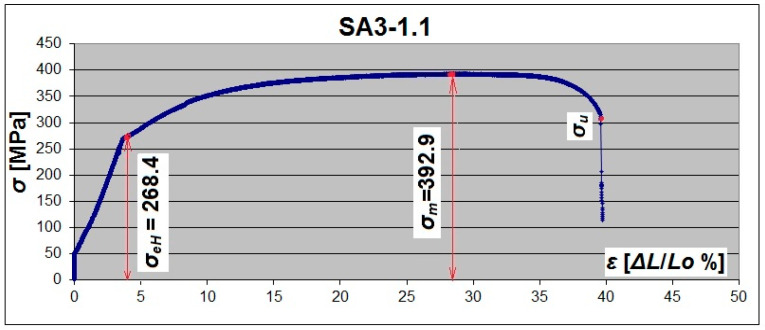
Tensile σ-ε diagram of a 3 mm thick arch steel sample with a corrugated web.

Figure 13 and Figure 14 shows reference diagrams for the flat and arched specimens with a thickness of 3 mm. The proportional limit, the yield strength *R_e_*, and the tensile strength *R_m_* are marked.

**Figure 15:**
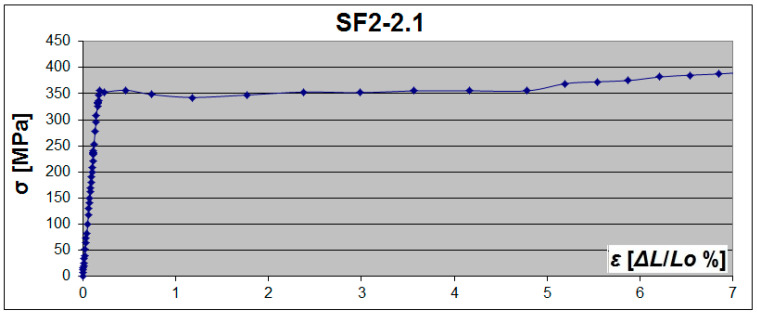
Examples of σ-ε tensile diagrams of flat steel samples obtained in DIC.

For flat specimens (SF), clear yield strength and a large flow area were observed—Figure 13 and Figure 15. There was practically no flow area in some arched specimens (SA). Only the diagram depression can be noticed, which illustrates the yield point (Figure 14).

**Figure 16:**
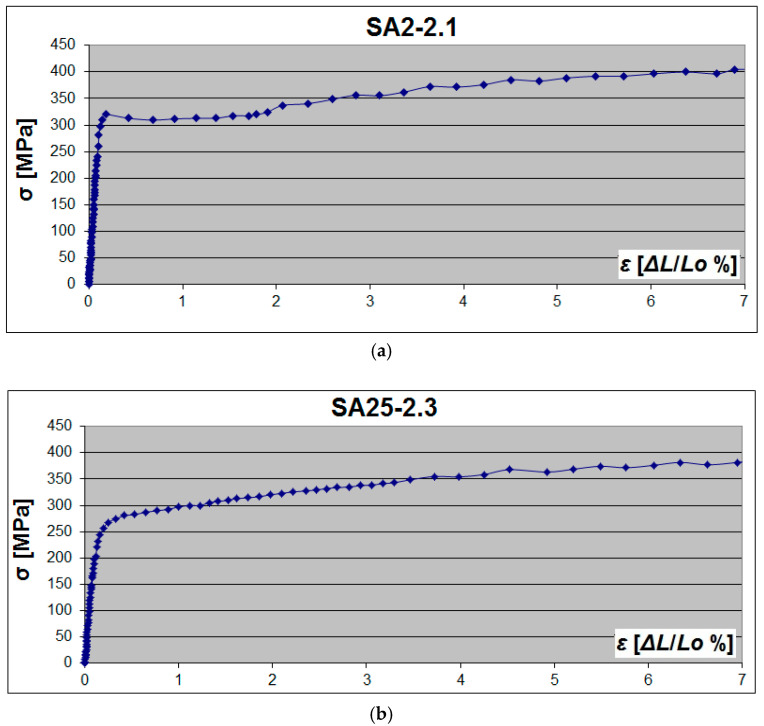
Examples of σ-ε tensile diagrams of arch steel samples obtained in DIC: (**a**) 2 mm arch sample; (**b**) 2.5 mm arch sample.

A yield plateau was observed in specimens from the SA2 series—Figure 16a. In the SA3 series, only 11 out of 24 specimens had an upper yield point *R_eH_*, and the other 13 specimens demonstrated only a flat, short-yield plateau with a slightly noticeable transition into the strengthening region. In all specimens from the series SA2.5, the yield plateau completely disappeared (the flow area of the material practically did not occur), but there was a rather short transition (the noticeable depression in the diagram representing the yield point) between the elastic region and the strengthening region—Figure 16b.

Material from webs with different thicknesses demonstrated different behaviour in the plastic region. The length of the yield plateau in the flat specimens was ~3.9% on average for the sheet with a thickness of 2 mm (SF2), ~1.4% for the sheet with a thickness of 2.5 mm (SF2.5), and ~2.8% for the sheet with a thickness of 3 mm (SF3). The length of the yield plateau in the arched specimens was reduced and on average equal to 1.8% for the specimens with a thickness of 2 mm, and 0.6% for the specimens with a thickness of 3 mm. The yield plateau completely disappeared in the specimens with a thickness of 2.5 mm. Hence, the mean reduction of the yield plateau length was 2.1% (SA2), at least 1.4% (SA2.5), and 2.2% (SA3).

The lack of clear yield point in the arched specimens or the shortened yield plateau and an increased tensile strength at simultaneously increased Young’s modulus indicated hardening of the material (Chapter 4), which is important considering the shear resistance of the corrugated web.

Corrugation of the sheet caused material hardening and broadening of the clear yield strength in the arched specimens. The range of elongation of both flat and arched specimens was similar and within an average range from 24 to 28%.

Mean values of deformation *εR_m_* determined with DIC and corresponding to the stress *R_m_* were similar for all series of the specimens, 17.1% on average for the specimens with a thickness of 2 and 3 mm, and 17.5% on average for the specimens with a thickness of 2.5 mm (at relatively small variation coefficients from 0.11 to 0.22). Differences in mean values between the arched and flat specimens of the same thickness were minor and did not exceed 0.24%. In addition, the overall (end) elongation of the specimens did not considerably differ within the specific thickness. Such results indicated the release of deformations restrained in the direction of the generating line and the compensation of deformations and stresses in the strengthening region.

## 3. Statistical Analysis of the Distribution of Mechanical Properties

The statistical analysis of examined specimens was performed to show the behaviour of mechanical properties along the curvature of the corrugated web. The parameters of normal distribution were applied for the probability density [31]. Variance *D^2^(R_e_)* and standard deviations *D(R_e_)* were determined for the yield strength and tensile strength of the tested specimens. For the determined parameters of the normal distribution of yield strength *R_e_* and tensile strength *R_m_*, characteristic values *R_ek_* and *R_mk_*, which are the bottom quantiles of 5% recommended in the standard EN 1990 [30], were obtained from Equation (1):

(1) Rek,mk=R¯e,m1−1.64VRe,m,

where *R_e,m_* is the mean value of yield strength or tensile strength *R_e_*, *R_m_*; *V_Re,m_* is the coefficient of variation of the yield strength *R_e_* or tensile strength *R_m_*.

The estimated coefficients of variation of both the yield strength and tensile strength demonstrate the deviation in the mean value. Thus, they are the preliminary image of the distribution of measured strength parameters for the tested specimen population.

Coefficients *V_Re,m_* obtained from the material tests were determined from the following equation:

(2) VRe,m=DRe,mR¯e,m,

where *D*(*R_e,m_)* is the standard deviation of yield strength *R_e_* or tensile strength *R_m_.*

The coefficients of yield strength and tensile strength *γ*_Me,m_, expressing the relationship between characteristic and design values resulting from transition of the calculation formula specified in the standard, were determined from the following equation:

(3) $$\gamma_{Me,m}=\frac{R_{ek,mk}}{{\bar{R}}_{e,m}-3.04D\left(R_{e,m}\right)},$$

where *γ*_Me,m_ is the coefficient of the yield strength *R_e_* or tensile strength *R_m_* and the design yield strength and tensile strength is $f_{y,u}={\bar{R}}_{e,m}-3.04D\left(R_{e,m}\right)$. The constant 3.04 is 0.8 × 3.8 and represents the sensitivity factor multiplied by the reliability index of structural elements for the resistance and the period of 50 years [30].

Coefficients of yield strength *γ_Me_* directly show the chosen safety level of the structure due to the applied material parameters.

Characteristic and design values, and variation and partial coefficients of yield strength and tensile strength are presented in Table 4 and Table 5.

**Figure 17:**
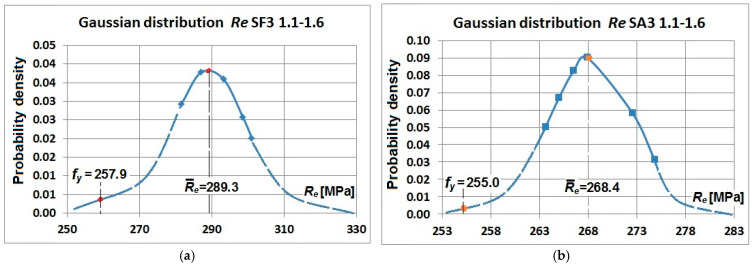
Normal distribution of the yield strength *R_e_* of samples cut from a corrugated web with a nominal thickness of 3 mm: (**a**) flat samples SF3-1.1–SF3-1.6 and (**b**) arch samples SA3-1.1–SA3-1.6.

**Figure 18:**
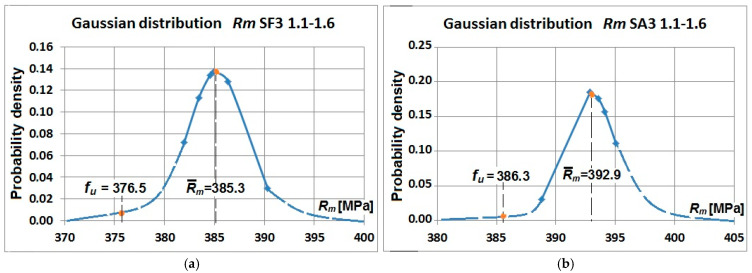
Normal distribution of the tensile strength Rm of samples cut from a corrugated web with a nominal thickness of 3 mm: (**a**) flat samples SF3-1.1–SF3-1.6 and (**b**) arch samples SA3-1.1–SA3-1.6.

Figure 17 and Figure 18 illustrate the examples of normal distributions of yield strength and tensile strength obtained for the flat and arched specimens cut out from corrugated webs with a thickness of 3 mm. These present the mean values of yield strength and tensile strength, and the design values.

**Figure 19:**
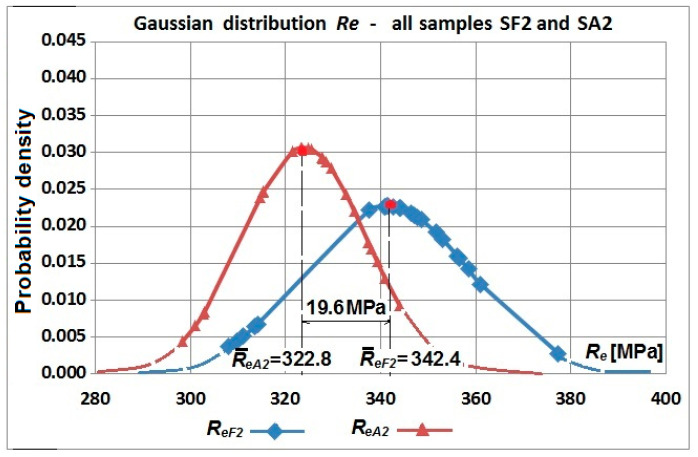
Normal distribution of yield strength Re samples cut from a corrugated web with a nominal thickness of 2 mm—flat samples SF2-1.1–SF2-1.6 and arch samples SA2-1.1–SA2-1.6.

**Figure 20:**
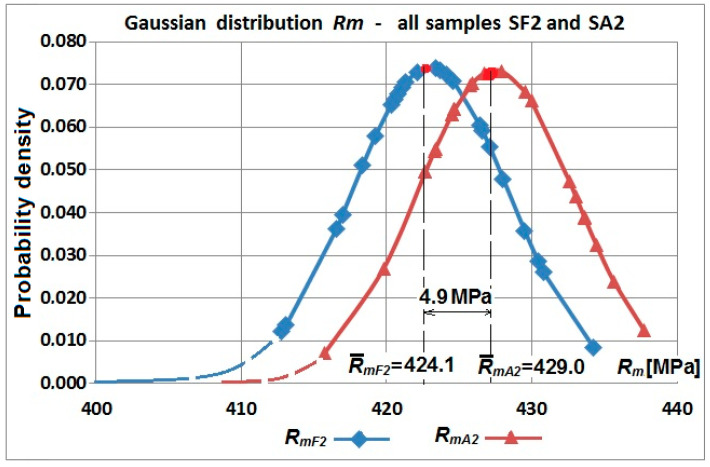
Normal distribution of tensile strength R_m_ samples cut from a corrugated web with a nominal thickness of 2 mm—flat samples SF2-1.1–SF2-1.6 and arch samples SA2-1.1–SA2-1.6.

However, the diagrams shown in Figure 17 and Figure 18 do not show changes in values of the yield strength against tensile strength as a whole. Yield strength of the flat specimens was greater than tensile strength of the arched specimens—Table 3 and Table 4. Hardening in the bending process resulted in greater values for the tensile strength of the arched specimens (Figure 19 and Figure 20).

Therefore, the range for yield strength and tensile strength in arched parts of the web wave was greater than in the flat part. The difference in the *R_m_*/*R_e_* ratio was up to 12% while only increasing tensile strength *R_m_* to 4%.

The minor variability in the variation coefficient *V_R_*, up to 0.05 for yield strength and up to 0.02 for tensile strength as obtained from the discussed tests, demonstrates a low distribution of strength parameters. This proves homogeneity of the material and a lack of impact of accidental perturbations on the random initiation of web buckling.

**Figure 21:**
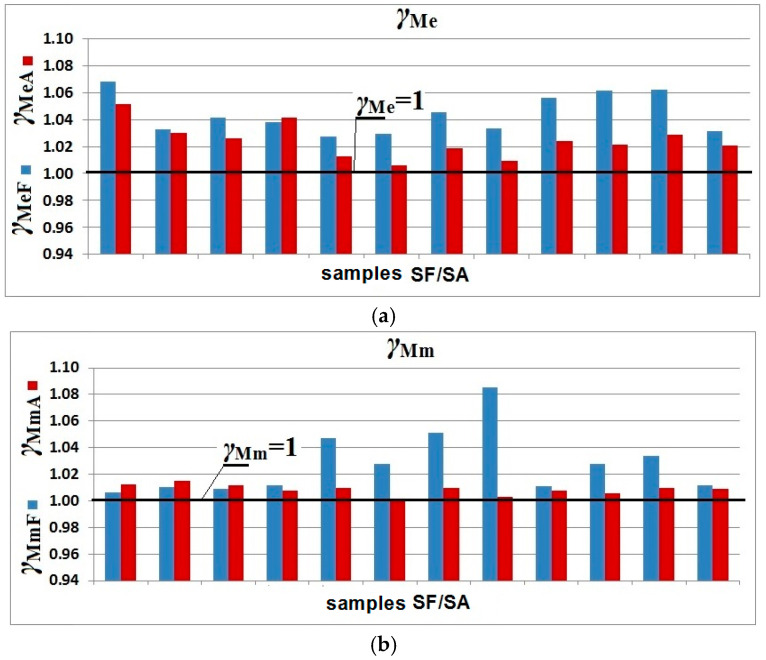
Coefficients *γ_M_* of flat and arched samples cut from a corrugated web: (**a**) yield strength *γ*_Me_ and (**b**) tensile strength *γ*_Mm_.

The above is confirmed by the obtained values for the partial coefficients of yield strength *γ_m_* and tensile strength, which are illustrated in Figure 21. The boundary line for the partial yield strength factor separating the characteristic value of yield strength from the design value is marked with a continuous line.

Coefficients of yield strength *γ*_Me_ and tensile strength *γ*_Mm_ obtained from the experimental tests are slightly greater than the coefficient *γ*_M_ = 1.0 specified in the European Standard [28]. This means that the standard EN 1990 [30] only marginally underrates the safety of steel structures, which does not affect the failure process. It should be emphasized that both yield strength and tensile strength *γ*_M_ of the arched specimens were closer to the standard value, that is, to one.

## 4. Hardness Tests

Because tests on mechanical parameters are required to be conducted only on the specimens cut out along the surface, and tensile tests gave values *R_e_* and *R_m_* only for average thicknesses of the specimen, a decision was made to perform supplementary hardness tests to assess possible hardening during bending of the sheet to a sinusoidal shape.

**Figure 22:**
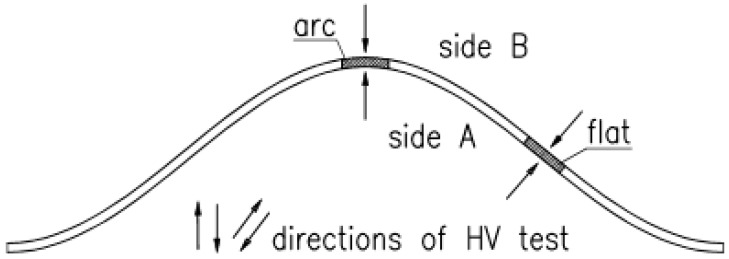
The position of the tested samples for HV hardness measurement before cutting them out. Arrows indicates direction of HV test.

**Figure 23:**
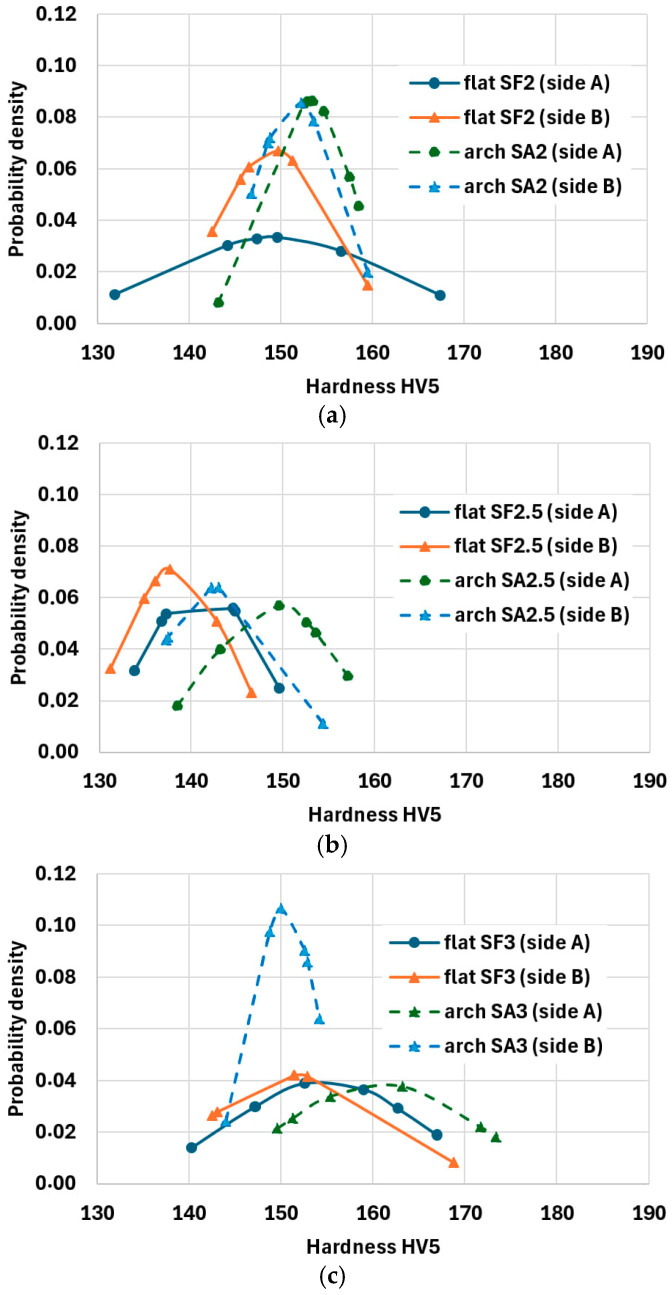
Probability density distributions of HV5 hardness of samples with different thicknesses: (**a**) 2 mm, (**b**) 2.5 mm, and (**c**) 3 mm.

Hardness tests were performed by the Vickers method on the specimens taken from three randomly selected sheets, one specimen from the flat fragment of corrugated sheet and one specimen from the arched fragment for each thickness (2, 2.5, and 3 mm) (Figure 22). In total, there were six specimens. The applied load was 5 kg (HV5). Surface hardness was tested on both sides of each specimen by conducting at least six marks. Sides A and B are shown in Figure 22. Mean values of hardness HV5 and indicators of variations are compared in Table 6, while distributions of probability density are illustrated in Figure 23.

A noticeable, yet minor difference in material hardness was observed for the specimens cut out from the flat and arched fragments, which confirmed variation of material features. Lower hardness values were found on side B (the convex side of the arched specimen or the flat specimen adjacent to the convex part of the corrugated sheet—cf. Figure 21). Differences between hardness measured on both sides of the specimens (A−B) were noticeably greater for the arched specimens; the thicker the material was, the greater these values were. Clear differences in hardness between sides A and B for the arched specimens could be related to a well-known phenomenon consisting in changes of results obtained from measured hardness depending on the sign and values of stress (in this case, residual stress). The obtained mean hardness value (A + B)/2 on both sides was greater from 2 to 4% for the arched specimens when compared to the flat ones. The average ratio of standard deviations *D_max_*/*D_min_* of hardness HV ranged from 1.026 to 1.034 for the flat specimens, and from 1.023 to 1.049 for the arched specimens.

**Figure 24:**
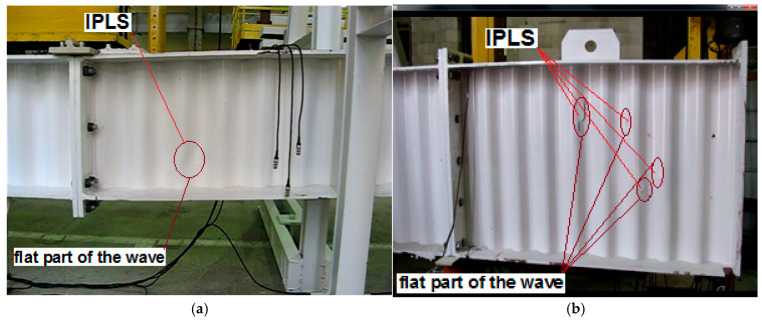
Initiation point loss of stability (IPLS) on flat sections of the web corrugation in girders with a corrugated web: (**a**) girder *h_w_* = 500 mm; (**b**) girder *h_w_* = 1250 mm [26].

Changes in hardness indicated the impact of the bending deformation of the arched specimens during the tests and its curvature on the obtained result. At the same time, the above confirmed the impact of sheet shape on initiating points of buckling (IPLS) in the corrugated web on its flat segments. Their presence was also demonstrated by the laboratory tests [23] which analysed 20 girders with a corrugated web having a height from 500 to 1500 mm. In all cases, initiating points of buckling were found in flat segments of the web corrugation, which led to local or global buckling of the corrugated web, and finally to failure of the girder. Figure 24a,b illustrate examples of girders at the initial phase of failure during the occurrence of initiating points of buckling, which unambiguously confirms close relations between the effect of the sheet shape of the web along the web curvature and the local increase in strength parameters. Due to this fact, design standards should be amended to determine the yield strength and critical shearing strength of corrugated web girders.

## 5. Values of Residual Deformations and Stresses

**Figure 25:**
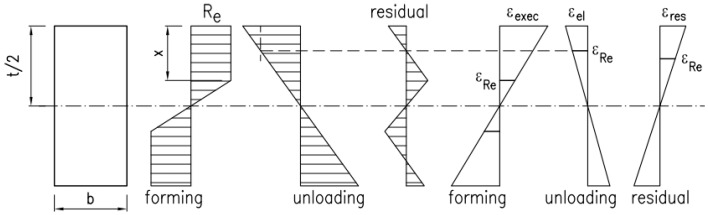
Formation of residual stresses and strains in the bending direction.

For maximum values of permanent deformation in the bending direction, mentioned in point 3, values of residual stress in that direction were theoretically estimated. Assuming that *x* is the height of the yield zone during the formation, and that the constitutive model is perfectly elastic and plastic, it can be expressed as follows (Figure 25):

(4) εexec=εRett−2x,

The perfectly elastic–plastic model does not take into account the strengthening of the material, which causes a slight deviation in the results compared to reality.

Residual stress is equal to the following:

(5) $$\varepsilon_{res}=\varepsilon_{exec}-\varepsilon_{el},$$

where *ε_el_* is the deformation under the load—Figure 25.

Assuming that the value of the plastic moment during the formation is equal to the moment of elastic relief, the following is obtained:

(6) $$M=R_{e}bx(t-x)+\frac{b{\left(t-2x\right)}^{2}}{6}R_{e}=\frac{bt^{2}}{6}E\varepsilon_{el}$$

After the transformation, the following equation is obtained:

(7) 4x3−6tx2−2εresεRet2x+εresεRet3=0

**Figure 26:**
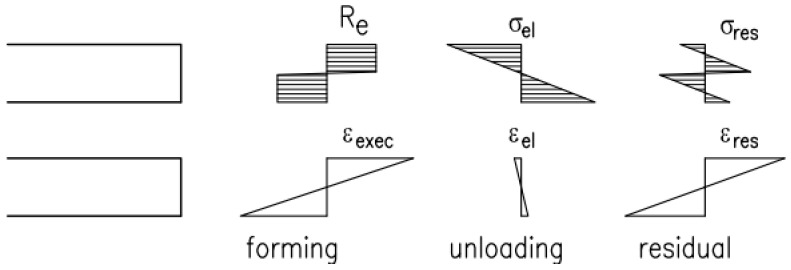
Strain and stress distributions according to the web result *t* = 2 mm.

The estimated reference distribution of residual stresses, in the direction of the bending of the sheet with a thickness of 2 mm, is presented in Figure 26. The analysis was based on the mean values of yield strength *R_e,mean_* obtained from the flat specimens (SF), while ε*_el_* = *R_e,mean_* /*E_mean_*. Stress values at the edge were approx. 50% *R_e_* for all thickness values.

Regardless of differences in the range of permanent deformations *ε_res_* determined with a web shape approximated with a sinus function, and more precise geometry, values of residual stress at the outer edge of the wave in a bending direction, determined with a perfectly elastic and plastic model, were equal to approx. 50% of yield strength *R_e_* (from 49.83 to 49.96%)—Table 7. 

Assuming that the flatness of the cross-section and the sinusoidal shape of the wave were kept, maximum values of deformations in the direction *s* (cf. Figure 4 and Figure 6b) for thicknesses of 2 mm, 2.5 mm, and 3 mm were 3.28%, 4.11%, and 4.93%, whereas for more realistic geometry with strictly flat and circular arch segments, these values were 2.56%, 3.18%, and 3.61%, respectively. A reduction in the length of the yield plateau was found to be greater than 50% of permanent deformation. As Poisson’s ratio cannot exceed 0.5, permanent deformation could not be the only factor affecting the behaviour of the specimens subjected to tension. It can be concluded that this result was considerably affected by stress compensation in the cross-section during the tensile test.

## 6. Conclusions

A small increase in the tensile strength of arched specimens was observed during the tests for all thickness values when compared to the flat specimens. What is more important from a practical point of view, is that ambiguous differences were noticed for yield strength, which was the base for determining the design strength of the material (regardless of the fact that yield strength was always clearly higher than that guaranteed by the manufacturer).

A minor increase in yield strength with reference to the flat specimens was found in the specimens of series SA2.5, and there was a drop of a few percentage points for the specimens of series SA2 and SA3. A change of that nature depends on the length and the type of yield plateau of the starting material (the flat specimen) in connection with permanent deformations forced during the formation of corrugated web and related residual stresses.

At the same time, an increase in Young’s modulus was observed for all three values for thickness, which had a positive effect on stability in the case of elastic buckling.

Mean differences in material hardness between specimens cut out from the flat and arched parts of the web were subtle, yet noticeable, ranging from 2% to 4% at the highest estimated deformation resulting from bending in the direction of the wave, and from 3.3% to 5.3% (assuming the wave shape according to a sinus function). Steel hardening due to strain effect was low, but sufficient for a slight increase in tensile strength *R_m_*.

Material from the flat specimens with all three values of thickness demonstrated a clear yield plateau, which indicated a subtle effect of deformations and free stresses created during the production step. Restrained deformations and free stresses in a direction perpendicular to bending (along the formed wave) were not sufficient for reaching the strengthening region of the sheets with a thickness of 2 mm and 3 mm.

Differences in elongation at the maximum load of the specimen (reached stress values *R_m_*) and at failure between the flat and the arched specimens were minor, which indicated the compensation of deformations and stresses in the specimen cross-section after exceeding the yield strength. It means that at sufficient ductility of steel, stresses and deformations in the strengthening region of the material could be compensated. Considering homogeneity of compressed and tensile fields, the mean (global) effect of free stresses on the obtained resistance of the specimen in a bending direction (the *x* axis) should not be regarded as significant.

The analysis of initiating points of buckling indicates that the initiated buckling (IPLS points) occurs within the area of flat segments of the webs. Apart from the effect of geometry, this paper also shows the possible effect of a change within the yield strength.

## 7. Limitations and Future Work

The presented tests were performed on a limited population of steel shipments and the only manufacturer in Poland. However, differentiation of strength parameters in the web was confirmed, and the technology of fabrication of SIN girders is similar in other countries. Currently/commonly used analytical models assume a steady value of yield strength within the whole corrugated web. The authors anticipate that in the next stage of their work, they will perform a theoretical analysis of the web stability taking into account differentiation of strength parameters.

## Figures and Tables

**Table 1:** The investigations included.

Number of Flat Sample	Web *h_w_ x t_w_* [mm]	Number of Arch Sample	Web *h_w_ x t_w_* [mm]
1	2	1	2
SF2-1.1–SF2-1.6	333 × 2	SA2-1.1–SA2-1.6	333 × 2
SF2-2.1–SF2-2.6	333 × 2	SA2-2.1–SA2-2.6	333 × 2
SF2-3.1–SF2-3.6	333 × 2	SA2-3.1–SA2-3.6	333 × 2
SF2-4.1–SF2-4.6	333 × 2	SA2-4.1–SA2-4.6	333 × 2
SF2.5-1.1–SF2.5-1.6	333 × 2.5	SA2.5-1.1–SA2.5-1.6	333 × 2.5
SF2.5-2.1–SF2.5-2.6	333 × 2.5	SA2.5-2.1–SA2.5-2.6	333 × 2.5
SF2.5-3.1–SF2.5-3.6	333 × 2.5	SA2.5-3.1–SA2.5-3.6	333 × 2.5
SF2.5-4.1–SF2.5-4.6	333 × 2.5	SA2.5-4.1–SA2.5-4.6	333 × 2.5
SF3-1.1–SF3-1.6	333 × 3	SA3-1.1–SA3-1.6	333 × 3
SF3-2.1–SF3-2.6	333 × 3	SA3-2.1–SA3-2.6	333 × 3
SF3-3.1–SF3-3.6	333 × 3	SA3-3.1–SA3-3.6	333 × 3
SF3-4.1–SF3-4.6	333 × 3	SA3-4.1–SA3-4.6	333 × 3

F—flat sample, A—arch sample.

**Table 2:** Summary of parameters for testing the yield strength, tensile strength and Young’s modulus of flat samples taken from a corrugated web.

Number of Flat Sample	${\bar{S}}_{0}$	${\bar{L}}_{0}$	${\bar{L}}_{u}$	${\bar{A}}_{10}$	${\bar{P}}_{eH}$	${\bar{P}}_{m}$	${\bar{R}}_{e}$	${\bar{R}}_{m}$	${\bar{R}}_{m}/{\bar{R}}_{e}$	$\bar{E}$
	mm^2^	mm	mm	%	kN	kN	MPa	MPa		MPa
SF2-1.1–SF2-1.6	40.2	70.0	97.0	27.3	13.0	16.7	323.6	415.2	1.28	216.6
SF2-2.1–SF2-2.6	40.8	70.0	98.4	25.9	14.2	17.3	346.6	422.6	1.22	211.0
SF2-3.1–SF2-3.6	40.2	70.0	98.4	25.7	14.2	17.3	353.5	430.5	1.22	218.0
SF2-4.1–SF2-4.6	40.4	70.0	97.1	24.7	14.0	17.1	346.0	423.5	1.22	210.6
Mean values for 2 mm flat samples							342.4	424.1	1.24	214.0
SF2.5-1.1–SF2.5-1.6	51.2	80.0	110.2	24.2	13.3	19.7	259.8	383.7	1.48	190.3
SF2.5-2.1–SF2.5-2.6	51.0	80.0	114.5	27.6	13.4	20.6	262.5	403.6	1.54	205.0
SF2.5-3.1–SF2.5-3.6	51.0	80.0	112.0	26.5	13.4	20.3	258.6	392.7	1.52	201.8
SF2.5-4.1–SF2.5-4.6	51.9	80.0	115.6	25.4	13.2	19.9	254.6	383.0	1.50	199.6
Mean values for 2.5 mm flat samples							258.9	390.8	1.51	199.2
SF3-1.1–SF3-1.6	60.3	90.0	122.8	24.3	17.4	23.2	289.3	385.3	1.33	203.9
SF3-2.1–SF3-2.6	61.7	90.0	123.9	25.1	17.8	23.6	289.2	381.9	1.32	211.3
SF3-3.1–SF3-3.6	60.0	90.0	124.3	23.8	17.5	22.9	291.4	381.4	1.31	203.4
SF3-4.1–SF3-4.6	62.2	90.0	123.6	24.9	17.9	23.4	288.5	376.2	1.30	172.0
Mean values for 3 mm flat samples							289.6	381.2	1.32	206.3

**Table 3:** Summary of parameters for testing the yield strength, tensile strength, and Young’s modulus of arch samples taken from a corrugated web.

Number of Arch Sample	${\bar{S}}_{0}$	${\bar{L}}_{0}$	${\bar{L}}_{u}$	${\bar{A}}_{10}$	${\bar{P}}_{eH}$	${\bar{P}}_{m}$	${\bar{R}}_{e}$	${\bar{R}}_{m}$	${\bar{R}}_{m}/{\bar{R}}_{e}$	$\bar{E}$
	mm^2^	mm	mm	%	kN	kN	MPa	MPa		MPa
1	2	3	4	5	6	7	8	9	10	11
SA2-1.1–SA2-1.6	41.0	70.0	99.0	26.3	12.6	17.3	307.9	422.8	1.37	224.4
SA2-2.1–SA2-2.6	40.7	70.0	97.6	25.1	13.3	17.5	327.9	429.5	1.31	222.2
SA2-3.1–SA2-3.6	40.7	70.0	97.1	24.7	13.1	17.5	322.0	429.3	1.33	214.9
SA2-4.1–SA2-4.6	40.7	70.0	99.1	23.6	13.6	17.7	335.3	435.5	1.30	224.5
Mean values for 2 mm arch samples							322.8	429.0	1.33	221.3
SA2.5-1.1–SA2.5-1.6	50.7	80.0	114.9	28.0	13.6	20.4	267.4	401.9	1.50	205.1
SA2.5-2.1–SA2.5-2.6	50.7	80.0	115.5	28.4	13.4	20.1	264.5	397.5	1.50	208.2
SA2.5-3.1–SA2.5-3.6	50.7	80.0	112.5	26.0	13.8	20.6	272.9	405.7	1.49	201.9
SA2.5-4.1–SA2.5-4.6	50.7	80.0	114.3	27.4	13.5	20.2	266.2	398.9	1.50	212.4
Mean values for 2.5 mm arch samples							267.7	400.8	1.50	206.9
SA3-1.1–SA3-1.6	61.0	90.0	127.7	27.5	16.4	24.0	268.4	392.9	1.46	225.5
SA3-2.1–SA3-2.6	61.0	90.0	126.0	26.6	16.5	24.0	270.9	394.0	1.45	226.8
SA3-3.1–SA3-3.6	60.7	90.0	124.0	25.2	17.0	24.0	279.5	395.3	1.41	225.0
SA3-4.1–SA3-4.6	60.9	90.0	122.5	24.2	17.0	23.8	279.4	390.1	1.40	229.8
Mean values for 3 mm arch samples							274.5	393.1	1.43	226.8

**Table 4:** Normal distribution parameters of yield strength *R_e_* and tensile strength *R_m_* of flat samples cut from a corrugated web.

Number of Flat Sample	*R_ek_* [MPa]	*f_y_* [MPa]	*V_Re_*	*γ_Me_*	*R_mk_* [MPa]	*f_u_* [MPa]	*V_Rm_*	*γ_Mm_*
1	2	3	4	5	6	7	8	9
SF2-1.1–SF2-1.6	301.1	281.9	0.0424	1.0682	412.2	409.7	0.0043	1.0062
SF2-2.1–SF2-2.6	334.3	323.7	0.0217	1.0326	417.5	413.2	0.0073	1.0104
SF2-3.1–SF2-3.6	337.8	324.4	0.0270	1.0412	426.1	422.3	0.0063	1.0089
SF2-4.1–SF2-4.6	331.7	319.5	0.0252	1.0382	417.8	412.9	0.0082	1.0118
Values for all 2 mm flat samples *	318.0	297.1	0.0435	1.0702	414.7	406.7	0.0135	1.0196
SF2.5-1.1–SF2.5-1.6	251.9	245.1	0.0186	1.0276	364.5	348.2	0.0305	1.0470
SF2.5-2.1–SF2.5-2.6	254.0	246.8	0.0196	1.0292	391.2	380.6	0.0188	1.0279
SF2.5-3.1–SF2.5-3.6	246.1	235.5	0.0294	1.0452	371.6	353.6	0.0327	1.0509
SF2.5-4.1–SF2.5-4.6	245.3	237.4	0.0222	1.0334	350.8	323.4	0.0512	1.0850
Values for all 2.5 mm flat samples *	248.6	239.9	0.0241	1.0364	365.6	344.2	0.0392	1.0623
SF3-1.1–SF3-1.6	272.3	257.9	0.0357	1.0560	380.5	376.5	0.0075	1.0108
SF3-2.1–SF3-2.6	270.8	255.2	0.0387	1.0613	370.1	360.0	0.0188	1.0279
SF3-3.1–SF3-3.6	272.7	256.7	0.0392	1.0623	367.3	355.2	0.0226	1.0339
SF3-4.1–SF3-4.6	278.7	270.3	0.0208	1.0311	371.2	366.9	0.008	1.0117
Values for all 3 mm flat samples *	274.2	261.1	0.0324	1.0502	370.5	361.4	0.0170	1.0252

* Final values refer to all values calculated based on the normal distribution.

**Table 5:** Normal distribution parameters of yield strength *R_e_* and tensile strength *R_m_* of arch samples cut from a corrugated web.

Number of Arch Sample	*R_ek_* [MPa]	*f_y_* [MPa]	*V*_Re_	*γ_Me_*	*R_mk_* [MPa]	*f_u_* [MPa]	*V*_Rm_	*γ_Mm_*
1	2	3	4	5	6	7	8	9
SA2-1.1–SA2-1.6	291.1	276.8	0.0332	1.0517	416.9	411.8	0.0086	1.0123
SA2-2.1–SA2-2.6	317.0	307.7	0.0203	1.0303	422.0	415.7	0.0106	1.0153
SA2-3.1–SA2-3.6	312.6	304.6	0.0178	1.0263	423.6	418.7	0.0081	1.0117
SA2-4.1–SA2-4.6	320.4	307.8	0.0270	1.0412	431.6	428.3	0.0054	1.0077
Values for all 2 mm arch samples *	302.0	284.3	0.0392	1.0622	419.8	412.0	0.0130	1.0190
SA2.5-1.1–SA2.5-1.6	263.5	260.1	0.0089	1.0129	397.3	393.3	0.0070	1.0100
SA2.5-2.1–SA2.5-2.6	262.6	260.9	0.0044	1.0063	396.7	395.9	0.0014	1.0019
SA2.5-3.1–SA2.5-3.6	267.1	262.2	0.0128	1.0187	401.3	397.5	0.0066	1.0095
SA2.5-4.1–SA2.5-4.6	263.4	261.0	0.0064	1.0091	397.6	396.5	0.0020	1.0029
Values for all 2.5 mm arch samples *	261.4	255.9	0.0145	1.0213	394.9	389.9	0.0090	1.0129
SA3-1.1–SA3-1.6	261.2	255.0	0.0164	1.0241	389.3	386.3	0.0055	1.0078
SA3-2.1–SA3-2.6	264.4	258.9	0.0145	1.0213	391.3	389.0	0.0042	1.0059
SA3-3.1–SA3-3.6	270.7	263.2	0.0191	1.0284	390.8	386.9	0.0070	1.0100
SA3-4.1–SA3-4.6	272.9	267.3	0.0142	1.0208	386.2	382.8	0.0061	1.0087
Values for all 3 mm arch samples *	263.8	254.6	0.0238	1.0360	388.3	384.3	0.0073	1.0105

* Final values refer to all values calculated based on the normal distribution.

**Table 6:** Mean values and hardness variation indices HV5.

Material Thickness	Sample (see Figure 21)	Side of the Web	Hardness HV5			Coefficient of Variation	R_e,mean_ ^1)^	R_e,mean_/ HV5_mean_	R_m,mean_ ^1)^	R_m,mean_/ HV5_mean_
			Mean	A − B	(A + B)/2					
-	-	-	-	-	-	%	MPa	-	MPa	-
2 mm	flat	A	149.5	0.3	149.4	7.99	341.5	2.24	423.0	2.83
		B	149.2			4.62		2.25		2.84
	arc	A	153.3	1.7	152.5	3.02	324.5	2.10	427.8	2.79
		B	151.6			3.05		2.12		2.82
2.5 mm	flat	A	141.2	3.0	139.7	4.30	261.9	1.84	392.7	2.78
		B	138.2			4.04		1.88		2.84
	arc	A	149.1	6.3	146.0	4.68	264.7	1.78	400.5	2.69
		B	142.8			4.36		1.85		2.81
3 mm	flat	A	154.8	3.1	153.3	6.47	289.9	1.87	380.1	2.45
		B	151.7			6.26		1.91		2.50
	arc	A	160.8	10.4	155.6	6.39	274.2	1.71	392.7	2.44
		B	150.4			2.47		1.83		2.61

^1)^ From all tensile tests at a given thickness.

**Table 7:** Estimation of residual strain and stress values (sinusoidal shape).

*t*	ε_max_ = ε_*res*_	${\bar{R}}_{e}$	$\bar{E}$	ε_Re_	x	σ_*res*_ ^1)^
mm	-	MPa	GPa	-	mm	MPa
2	0.03286	341.4	214.0	0.001595	0.955	170.4
2.5	0.04108	262.2	199.2	0.001316	1.212	131.0
3	0.05299	289.9	206.3	0.001405	1.462	144.9

^1)^ On the edge.

## Data Availability

The original contributions presented in this study are included in the article. Further inquiries can be directed to the corresponding author.
